# A dual-aptazyme system for genetic complementation reveals stage-specific roles for the *Trypanosoma cruzi* cytoskeleton-associated protein 5.5

**DOI:** 10.1128/msphere.00090-26

**Published:** 2026-05-29

**Authors:** Justin Wiedeman, Ganesh Babu Malli Mohan, Gonzalo Seminario-Mondejar, Ronald Drew Etheridge

**Affiliations:** 1 Center for Tropical and Emerging Global Diseases, University of Georgia 1355, Athens, Georgia; 2 Department of Cellular Biology, University of Georgia 1355, Athens, Georgia; Virginia-Maryland College of Veterinary Medicine 70732, Blacksburg, Virginia, USA

**Keywords:** *Trypanosoma cruzi*, Chagas disease, cytoskeleton, CAP5.5, conditional knockdown, shark, cell morphogenesis, cytokinesis, fluorescent image analysis

## Abstract

Parasitic trypanosomatids, such as *Trypanosoma cruzi*, *Trypanosoma brucei*, and *Leishmania spp*., cause devastating tropical diseases, with *T. cruzi* being increasingly recognized as a public health concern in the United States due to established sylvatic cycles and rising autochthonous transmission. Although *T. brucei* has long served as the model trypanosomatid due to the array of molecular tools available for its study, *T. cruzi* shares greater biological similarity with the majority of trypanosomatids regarding nutrient uptake, transmission strategy, and metabolism. Recent advancements in genetic tools have now enabled in-depth studies in *T. cruzi* previously restricted to *T. brucei*. Here, using the Small Hammerhead Aptazyme-Regulated Knockdown (SHARK) system, we report the characterization of cytoskeleton-associated protein 5.5 (CAP5.5) in *T. cruzi*. To validate this phenotype, we introduce a novel method for simultaneous knockdown and rescue of a target protein using two tetracycline-responsive aptazymes. Knockdown of CAP5.5 revealed stage-specific roles in the maintenance of cell shape, organelle segregation, and proliferation. To quantify these phenotypes, we leveraged open-source, state-of-the-art segmentation software to develop a semi-automated, high-throughput image analysis pipeline. Our data suggest a potential role for CAP5.5 in repairing motility-related damage to the microtubule cytoskeletal array. These findings demonstrate the emerging utility of *T. cruzi* as a robust model trypanosomatid.

**IMPORTANCE:**

*Trypanosoma cruzi* is the causative agent of Chagas disease, a neglected tropical disease that presents a growing public health concern. Historically, research into *T. cruzi* biology has been hindered by a scarcity of genetic tools, forcing scientists to rely on models from related but biologically distinct parasites. In this study, we utilize genetic tools (the SHARK conditional knockdown system) to characterize a cytoskeletal protein, CAP5.5, revealing that it is required for the parasite to maintain its shape and divide during its motile stage. We further demonstrate the use of a dual-control system that allows simultaneous gene knockdown and rescue, alongside a new high-throughput 3D imaging pipeline. This work establishes a rigorous technical framework that empowers the community to conduct in-depth investigations of essential *T. cruzi* genes directly, thus accelerating the discovery of potential therapeutic targets.

## INTRODUCTION

Named for their distinctive mitochondrial organelle, the kinetoplastids are a diverse group of both free-living and parasitic protozoans. While valuable as models for divergent eukaryotic biology, they are also the causative agents of devastating diseases in humans and animals worldwide. Of the parasitic kinetoplastids, often termed trypanosomatids, three vector-borne parasites are responsible for major neglected tropical diseases: *T. cruzi*, *T. brucei*, and *Leishmania spp*., resulting in Chagas disease, African sleeping sickness, and leishmaniasis, respectively.

Historically, *T. brucei* has served as the *de facto* model for the group, owing largely to its amenability to axenic culture, well-annotated genome, and comprehensive genetic toolkit. However, this primacy overlooks a critical reality: *T. brucei* is a biological outlier among the vast majority of trypanosomatids. It lacks key ancestral traits shared among other trypanosomatids that are present in *T. cruzi,* such as the presence of a cytostome-cytopharynx complex (SPC) endocytic apparatus (1), a fecal transmission strategy (2, 3), and various conserved metabolic pathways such as histidine catabolism (4). Consequently, the utility of *T. brucei* as a representative model for general kinetoplastid biology is fundamentally constrained. Despite its disease relevance, *T. cruzi* research has historically trailed behind *T. brucei* due to significant technical barriers, specifically the lack of a high-quality annotated genome, low transfection efficiency, and the inability to conditionally regulate essential gene expression. However, recent improvements to genome sequencing (5), the introduction of CRISPR/Cas9 (6, 7), and the development of the first conditional gene knockdown system in *T. cruzi* (8) have relieved many of these limitations. These advances now make it possible to systematically interrogate *T. cruzi* biology at the molecular level, thus establishing it as a tractable model for kinetoplastids.

Like many protozoa (9), trypanosomes possess a subpellicular microtubule array, a corset-like structure arranged in a helical pattern that defines the cell’s shape and provides structural rigidity (10). These microtubules are polarized, oriented with their plus ends (growing ends) directed toward the posterior of the cell. Remarkably, the array maintains a constant inter-microtubule distance even as the cell body tapers at the poles due to selective microtubule termination as the cell narrows, allowing the remaining filaments to converge without overcrowding (10). Despite its structural role, the array is also highly dynamic. It undergoes continuous remodeling to accommodate the physical stress of motility, facilitate daughter-cell biogenesis, and support the drastic morphological changes associated with life-cycle transitions (11, 12). During the cell cycle, new microtubules are added to the array via intercalation between existing filaments (13) and insertion between the separating flagella of the mother and daughter cells (14). To date, approximately 20 proteins have been identified that localize to this essential cytoskeletal scaffold (11).

Among the proteins associated with the subpellicular array, the “calpain-like” family is particularly notable. Relative to other eukaryotes, kinetoplastids contain a highly expanded gene family encoding these proteins (15). However, unlike prototypical eukaryotic calpains, which are calcium-dependent cysteine proteases containing a catalytic Cys-His-Arg motif (16), most kinetoplastid homologs lack these essential residues and are thus classified as non-enzymatic “calpain-like” proteins. A prominent member of this family is the cytoskeleton-associated protein 5.5 (CAP5.5). In *T. brucei*, CAP5.5 is an essential component of the microtubule array required for parasite proliferation and cell morphogenesis (17). *T. brucei* encodes two developmentally regulated isoforms: TbCAP5.5 in procyclics and TbCAP5.5V in bloodstream forms (18). In contrast, *T. cruzi* possesses only a single CAP5.5 ortholog (19), the function of which remains entirely uncharacterized throughout the parasite’s life cycle.

In this study, we combine novel genetic tools with advanced semi-automated image analysis to functionally characterize TcCAP5.5, revealing critical stage-specific roles for this cytoskeletal protein in *T. cruzi*. Using the Small Hammerhead Aptazyme-Regulated Knockdown (SHARK) system (8), we achieved conditional depletion of TcCAP5.5 in both epimastigotes and intracellular amastigotes, uncovering distinct stage-specific phenotypes. We further validated these findings by developing a genetic complementation system that utilizes two distinct aptazymes to knock down endogenous TcCAP5.5 while inducing an ectopic copy. To strictly quantify morphological defects, we employed 3D image segmentation, generating the first reported high-resolution cell volume measurements for *T. cruzi*. Collectively, these findings not only define the critical functions of TcCAP5.5 but also demonstrate a rigorous technical framework for future molecular studies in this neglected pathogen.

## RESULTS

### CAP5.5 shark1-Ty-tagging and expression

The long-standing absence of a functional conditional knockdown system for *Trypanosoma cruzi* has crippled investigations of essential genes. The SHARK system (8) provided the first tools for conditional knockdown in *T. cruzi* and enabled simple functional characterization of essential genes. We sought to extend the utility of the SHARK system by also demonstrating its ability to simplify the validation of antibodies raised against putative essential genes. As a proof of principle, we directed our efforts toward characterizing the *T. cruzi* ortholog of the *Trypanosoma brucei* cytoskeleton-associated protein 5.5 isoforms (TbCAP5.5/TbCAP5.5V) (17), which are essential in both insect-stage procyclic and bloodstream form *T. brucei* (20).

**Fig 1:**
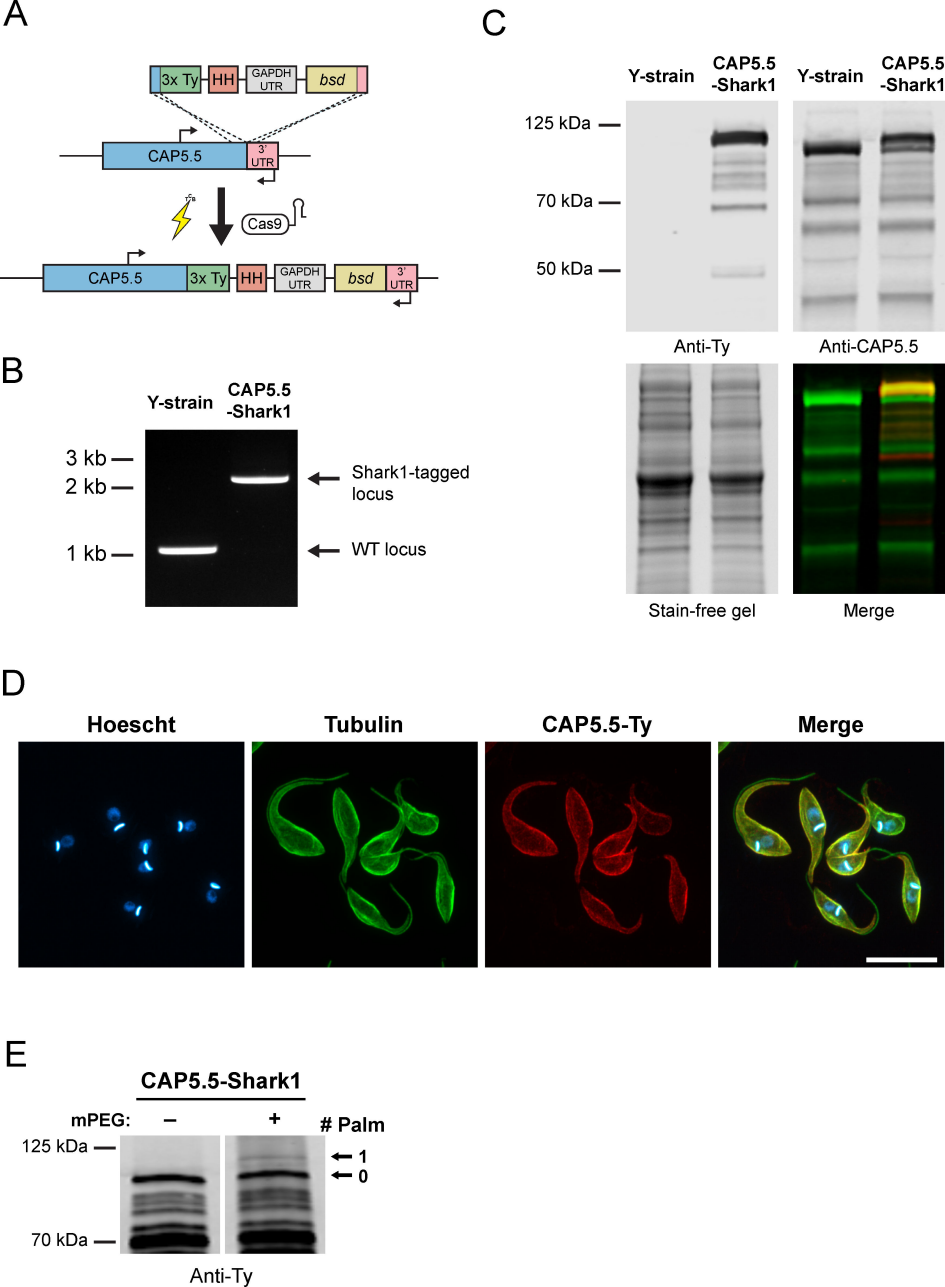
SHARK-tagging and localization of CAP5.5. (**A**) Schematic illustrating Cas9-directed integration of the Shark1 tag into the C-terminus of the endogenous CAP5.5 locus. The small directional arrows illustrate primer annealing sites. (**B**) Diagnostic PCR demonstrating tagging of the CAP5.5 locus. Expected parental (WT) locus amplicon = 1,036 bp. Expected tagged locus amplicon = 2,212 bp. (**C**) Western blot of CAP5.5-Shark1 cells labeled with anti-Ty (top left panel) or anti-CAP5.5 clone 3D8 (top right panel) antibodies. Stain-free gel illustrates protein loading before transfer. In the “Merge” panel, the anti-Ty signal is red, and the 3D8 signal is green. Expected MW for untagged CAP5.5 = 89.7 kDa and expected MW for 3x Ty-tagged CAP5.5 = 93.7 kDa. (**D**) Immunofluorescence microscopy of CAP5.5-Ty localization in epimastigotes. Trypanosomes were stained with Hoescht 33342 (DNA), TAT1 antibody (tubulin), and anti-Ty antibody (CAP5.5). Scale bar = 10 µm. (**E**) Acyl PEGyl exchange gel shift (APEGs) assay. Arrow (#1) highlights PEGyl-induced size shift due to single palmitoylation site conjugation to CAP5.5-Ty.

We first tagged TcCAP5.5 (TcYC6_0107360) (hereafter referred to as CAP5.5) at both of its endogenous loci with a C-terminal Shark1 aptazyme with a 3x-Ty epitope tag (Fig. 1A and 1B). A western blot using an anti-Ty antibody revealed a primary band near the expected molecular weight (MW) of CAP5.5-3xTy (93.7 kDa) along with multiple smaller bands (Fig. 1C). We raised three monoclonal antibodies against recombinant CAP5.5 (Fig. S1A) and compared reactivity to *T. cruzi* cell lysates from different discrete typing units (DTUs) (recently summarized in reference 21), including axenic cultures of recent field isolates (“Ts6” [DTU-I], from *Triatoma sanguisuga* insects collected in 2024 in Florida, USA, and “m20392” and “m21066” [both DTU IV] [22], from macaques in Texas, USA). All three antibodies cross-reacted with a primary band near 90 kDa (Fig. S1B). Interestingly, the 3D8 antibody clone, which cross-reacted with the highest affinity to Y-strain CAP5.5, did not cross-react with lysate in DTU-I strain epimastigotes (e.g., Brazil and Ts6, typing data not shown). In a dual-color fluorescent western blot combining anti-CAP5.5 clone 3D8 and anti-Ty antibodies, the anti-CAP5.5 signal (clone 3D8) overlapped with the anti-Ty signal (Fig. 1C).

Next, we investigated the subcellular localization of CAP5.5 by immunofluorescence (IFA) microscopy. The 3D8 antibody labeling was observed along the periphery of the cell body but not the flagellum (Fig. S1C), consistent with reports from *T. brucei* (17). As the IFA signal was generally faint using the 3D8 antibody, we also performed an IFA with an anti-Ty antibody and found a similar pattern of localization along the cortical cytoskeleton for CAP5.5-Ty (Fig. 1D).

To examine the structure of CAP5.5 in more detail, we generated a multiple sequence alignment of the two *T. brucei* CAP5.5 isoforms and their *T. cruzi* ortholog (Fig. S2). The *T. brucei* paralogs share 78.9% sequence identity, whereas each is approximately 47% identical to the *T. cruzi* ortholog. Despite high conservation within the central calpain-like domain, the *T. cruzi* ortholog diverges significantly from the *T. brucei* isoforms at both the N- and C-termini. Such structural divergence indicates the potential for species-specific binding partners or distinct regulatory mechanisms. Notably, similar to the *T. brucei* paralogs, the *T. cruzi* CAP5.5 protein possesses a conserved N-terminal dual acylation motif. This motif, previously shown to be both myristoylated and palmitoylated in *T. brucei*, likely functions to anchor the protein to the plasma membrane (17). To confirm the palmitoylation of CAP5.5 in *T. cruzi,* we used an acyl PEGyl exchange gel shift (APEGS) assay (23) where the presence of S-palmitoylated residues manifests as a shift in the observed molecular weight of the target protein when subjected to SDS-PAGE and western blotting. With CAP5.5, we observed a single band of increased weight of the protein, likely corresponding to a single palmitoylation site (Fig. 1E).

### CAP5.5 knockdown slows proliferation

**Fig 2:**
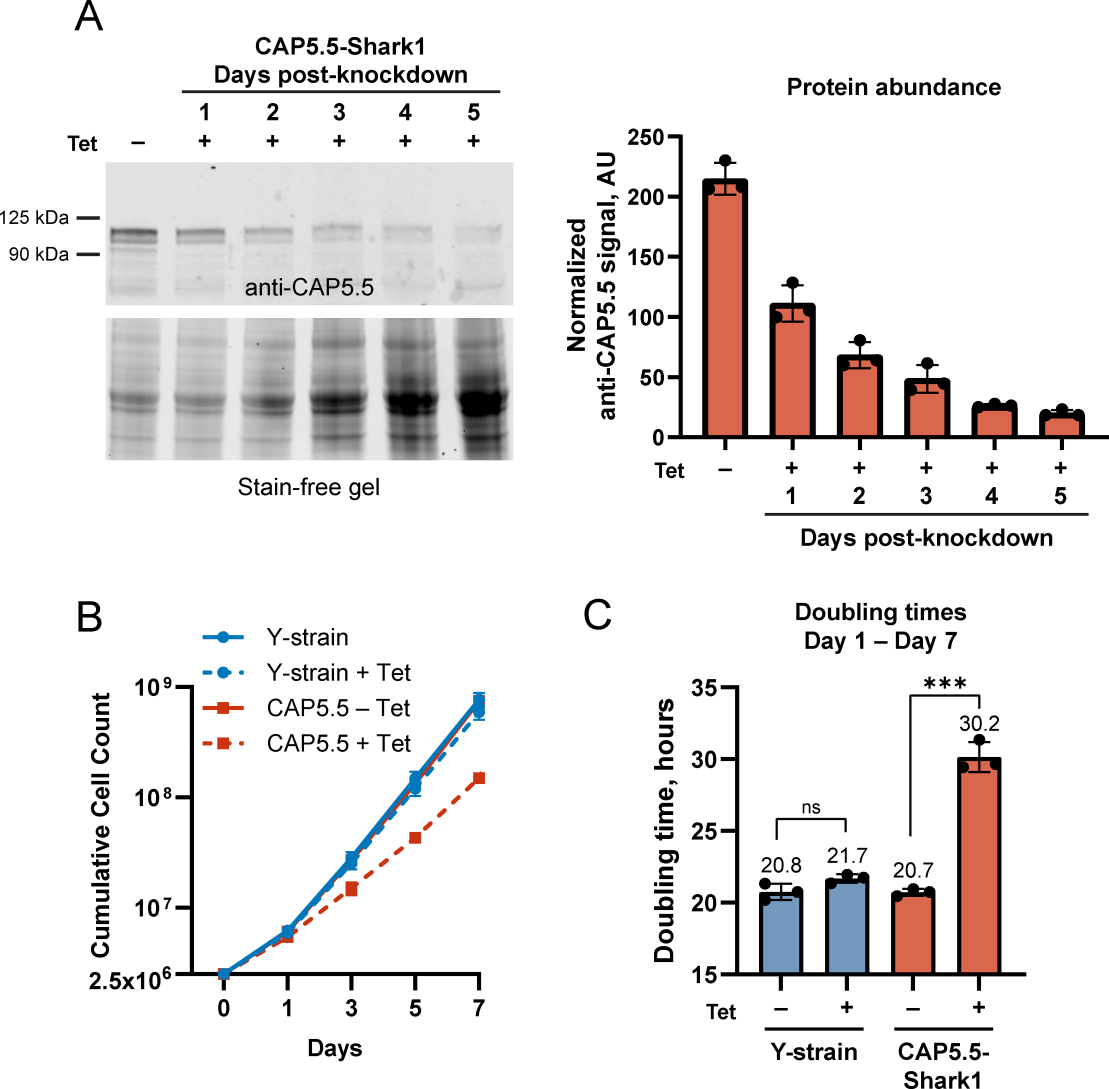
Knockdown of CAP5.5 using Shark1. (**A**) Left panel: Representative western blot time course of CAP5.5 protein abundance following tetracycline (Tet) addition. The membrane was labeled with anti-CAP5.5 3D8 antibody. Stain-free gel illustrates total protein loading before transfer. Right panel: Quantification of anti-CAP5.5 signal after knockdown from three independent replicates. Error bars represent std. dev. (**B**) Proliferation of Y-strain (parental line) and CAP5.5-Shark1 epimastigotes following Tet addition from three independent replicates. Error bars represent standard deviation (std. dev.). (**C**) Doubling times for Y-strain and CAP5.5-Shark1 epimastigotes calculated from data presented in B. Error bars represent std. dev. Statistical significance was assessed using Student’s T-test. For Y-strain, *P* = 0.07. For CAP5.5-Shark1, *P* = 1 × 10^−4^. ns = not significant. ***P = 0.0001.

RNAi against TbCAP5.5 is reported to slow proliferation in *Trypanosoma brucei* (20). To see whether CAP5.5 is necessary for proliferation in *T. cruzi* and to validate the specificity of the anti-CAP5.5 antibody (3D8), we induced CAP5.5 knockdown with tetracycline (Tet) in epimastigotes and measured protein expression (Fig. 2A) and parasite proliferation (Fig. 2B) over time. In CAP5.5-Shark1 cells, the average normalized CAP5.5 signal decreased by 91% after 5 days (Fig. 2A quantification, right panel), and the average doubling time increased from 20.7 h to 30.2 h (*P* = 1.1 × 10^−4^, Student’s T-test) (Fig. 2C). In contrast, in Y-strain (parental line) epimastigotes, Tet treatment had no significant effect on the average CAP5.5 signal (*P* = 0.08, Student’s T-test) (Fig. S2) or on doubling time (*P* = 0.07, Student’s T-test) after 5 days (Fig. 2C). Because the endogenous CAP5.5 signal overlaps with the Ty-epitope tagged CAP5.5 signal (Fig. 1C) and decreases after CAP5.5 knockdown (Fig. 2), we conclude that the 3D8 antibody specifically recognizes the parasite CAP5.5 protein. Furthermore, we conclude that CAP5.5 promotes robust epimastigote proliferation.

### CAP5.5 is required for normal cytokinesis and maintenance of cell body shape in epimastigotes

**Fig 3:**
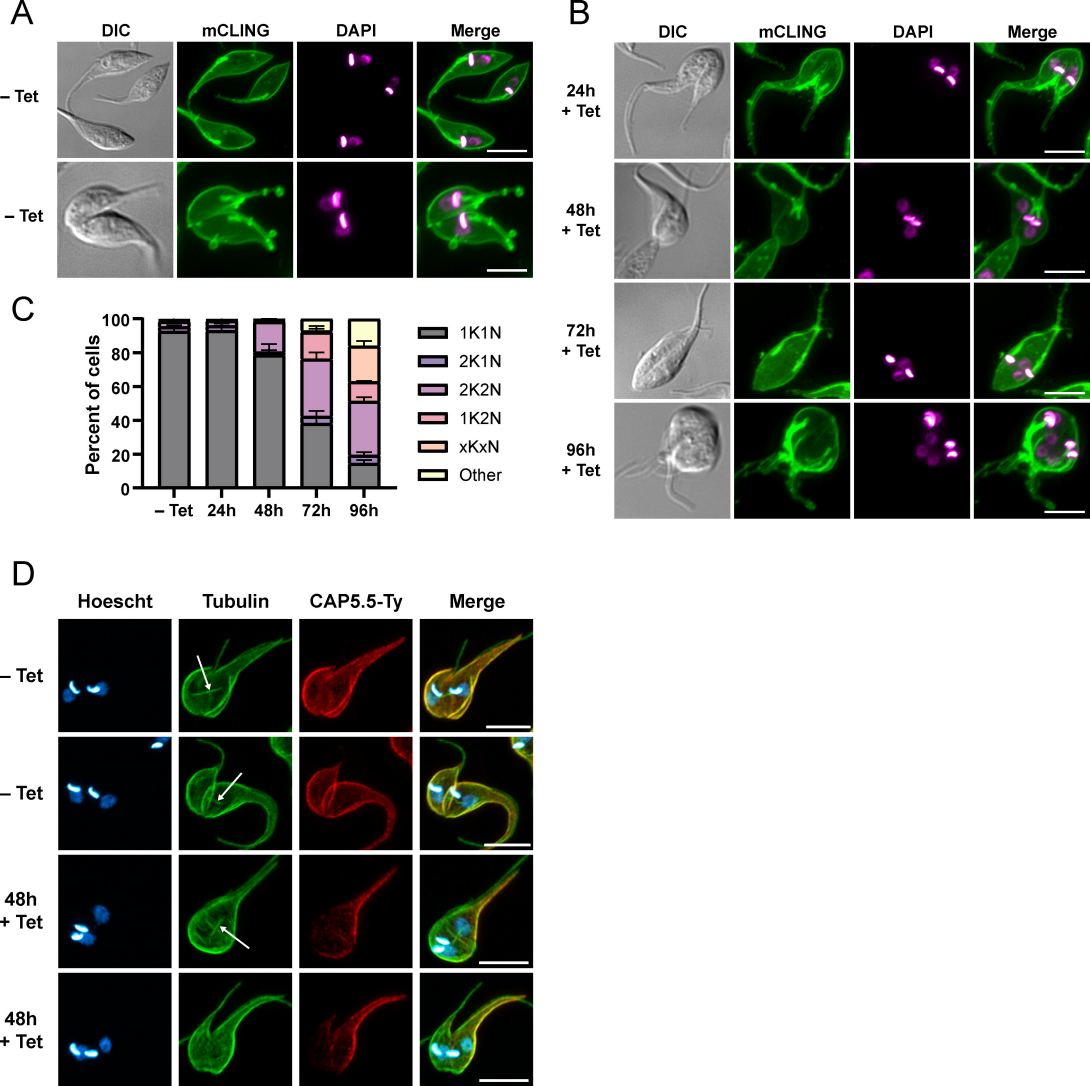
Effect of CAP5.5 knockdown on epimastigote morphology and kinetoplast and nucleus configuration. (**A**) Uninduced CAP5.5-Shark1 epimastigotes were stained with the membrane dye mCLING and the DNA dye DAPI. Top row: Pre-mitotic trypanosomes, each containing one kinetoplast (K) and one nucleus (N). Bottom row: Post-mitotic trypanosome containing 2K and 2N undergoing cytokinesis. (**B**) Post-mitotic CAP5.5-Shark1 epimastigotes were collected at the indicated time points after tetracycline (Tet) addition. (**C**) Quantification of the number of K and N per cell at the indicated time points after Tet addition. The average percentage of each cell type from three independent replicates is presented. Error bars represent std. dev. *N* > 150 trypanosomes at each time point. (**D**) Immunofluorescence microscopy of post-mitotic CAP5.5-Shark1 epimastigotes before (top two rows) and after (bottom two rows) Tet addition. Trypanosomes were stained with Hoescht 33342 (DNA), TAT1 antibody (tubulin), and anti-Ty antibody (CAP5.5). Arrows point to the mitotic spindle (if present). Scale bars = 10 µm.

Having demonstrated that CAP5.5 knockdown decreases epimastigote proliferation (Fig. 2C), we sought to functionally characterize CAP5.5 by investigating the knockdown phenotype. We induced Shark1-mediated knockdown and collected trypanosomes for fluorescence microscopy every 24 h for 4 days. Epimastigotes stained with the fluorescent membrane dye mCLING (24) displayed profound morphological defects beginning after 48 h of CAP5.5 knockdown. In some instances, the kinetoplasts fail to segregate after replication (Fig. 3B, second row), and in other instances, the nuclei fail to segregate after mitosis (Fig. 3B, third row). Additionally, 2K2N trypanosome abundance increases 5.6-fold after 48 h of CAP5.5 knockdown, comprising 17.9% of all cells (Fig. 3C). A fraction of 2K2N trypanosomes fails to execute cytokinesis and instead undergoes additional rounds of DNA synthesis and mitosis, producing cells containing at least two kinetoplasts and more than two nuclei (Fig. 3B, bottom row), for a total of 28.7% of the population after 96 h (Fig. 3C). Tet treatment of Y-strain epimastigotes has no effect on K/N configuration (8). These data are consistent with failed organelle segregation (e.g., kinetoplast and nucleus) resulting in inhibition of cytokinesis.

Immunofluorescence microscopy analysis revealed that Shark1-mediated knockdown of CAP5.5-Ty reduced the anti-Ty signal roughly along the longitudinal axis of the cell body (Fig. 3D), in a manner similar to the RNAi knockdown phenotype in *T. brucei* (20). After 48 h of knockdown, we observed significant depletion of CAP5.5-Ty near the dorsal (flagellum-containing) (25) and posterior (opposite exterior flagellum tip) regions of the cell, with faint anti-Ty signal remaining near the ventral and anterior regions of the cell body (in Fig. 3D, the intensity of the anti-Ty signal has been increased in the +Tet images to aid in visualization).

**Fig 4:**
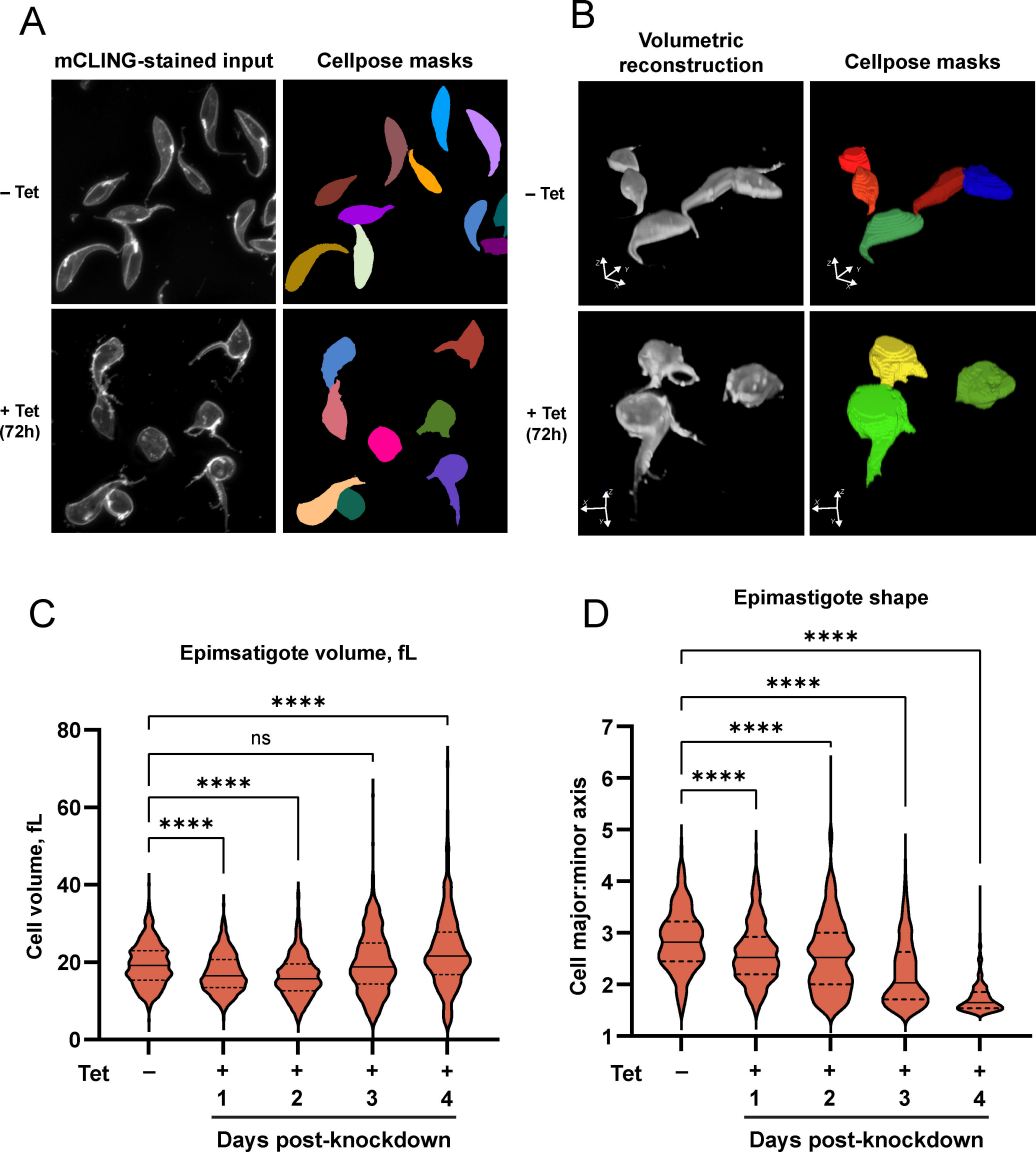
Semi-automated image masking and volumetric characterization of epimastigotes after CAP5.5 knockdown. (**A**) Maximum intensity projections of Z-stack images of CAP5.5-Shark1 epimastigotes stained with the membrane dye mCLING before (top row) or after (bottom row) tetracycline (Tet) addition. Images were segmented and cell masks created using Cellpose-SAM. (**B**) Volumetric reconstruction of Z-stack images of mCLING-stained epimastigotes before (top row) or after (bottom row) Tet addition. XYZ arrows in the lower left of each image indicate three-dimensional orientation relative to the original orientation of the acquired image. (**C**) Volume of 3D cell masks at the indicated time points after CAP5.5 knockdown. *N* > 353 cells for each time point. Statistical significance was assessed by comparing medians using a one-way ANOVA (*P* < 0.0001) with Holm-Sidak’s multiple comparisons test. For – Tet vs 24 h, *P* < 0.0001. For – Tet vs 48 h, *P* < 0.0001. For – Tet vs 72 h, *P* = 0.28. For – Tet vs 96 h, *P* < 0.0001. (**D**) Shape measurement (Major:Minor axis ratio) of 3D cell masks from the indicated time points after CAP5.5 knockdown. *N* > 353 cells at each time point. Statistical significance was assessed by comparing geometric means using a lognormal one-way ANOVA (*P* < 0.0001) with Dunnett’s multiple comparisons test. For – Tet vs 24 h, *P* < 0.0001. For – Tet vs 48 h, *P* < 0.0001. For – Tet vs 72 h, *P* < 0.0001. For – Tet vs 96 h, *P* < 0.0001. ****P < 0.0001; ns, not significant.

The loss of CAP5.5 dramatically alters epimastigote morphology (Fig. 3B). To determine the degree of these changes, we segmented Z-stack images of mCLING-stained (24) epimastigotes and created masks using a custom-trained segmentation model in Cellpose-SAM (26) (Fig. 4A and 4B). We then measured the volume (Fig. 4C) and shape (Fig. 4D) characteristics of the 3D masks using CellProfiler (27). CAP5.5 knockdown initially decreased the average cell volume potentially due to a loss of cytoskeletal integrity (i.e., compaction), but by day 4, cell volume increased from 19.4 fL to 22.9 fL (*P* < 0.0001, Holm-Sidak) (Fig. 4C). Next, we estimated the shape of each trypanosome by determining the length:width ratio (Major:Minor axis) of each parasite mask. After 4 days of knockdown, the average length:width ratio decreased from 2.9 to 1.75 (*P* < 0.0001, Dunnett’s), indicating an increase in overall roundness (Fig. 4D), consistent with observations in *T. brucei* after TbCAP5.5 knockdown (28).

### CAP5.5 promotes robust proliferation and maintains cell body shape in *T. cruzi* amastigotes

**Fig 5:**
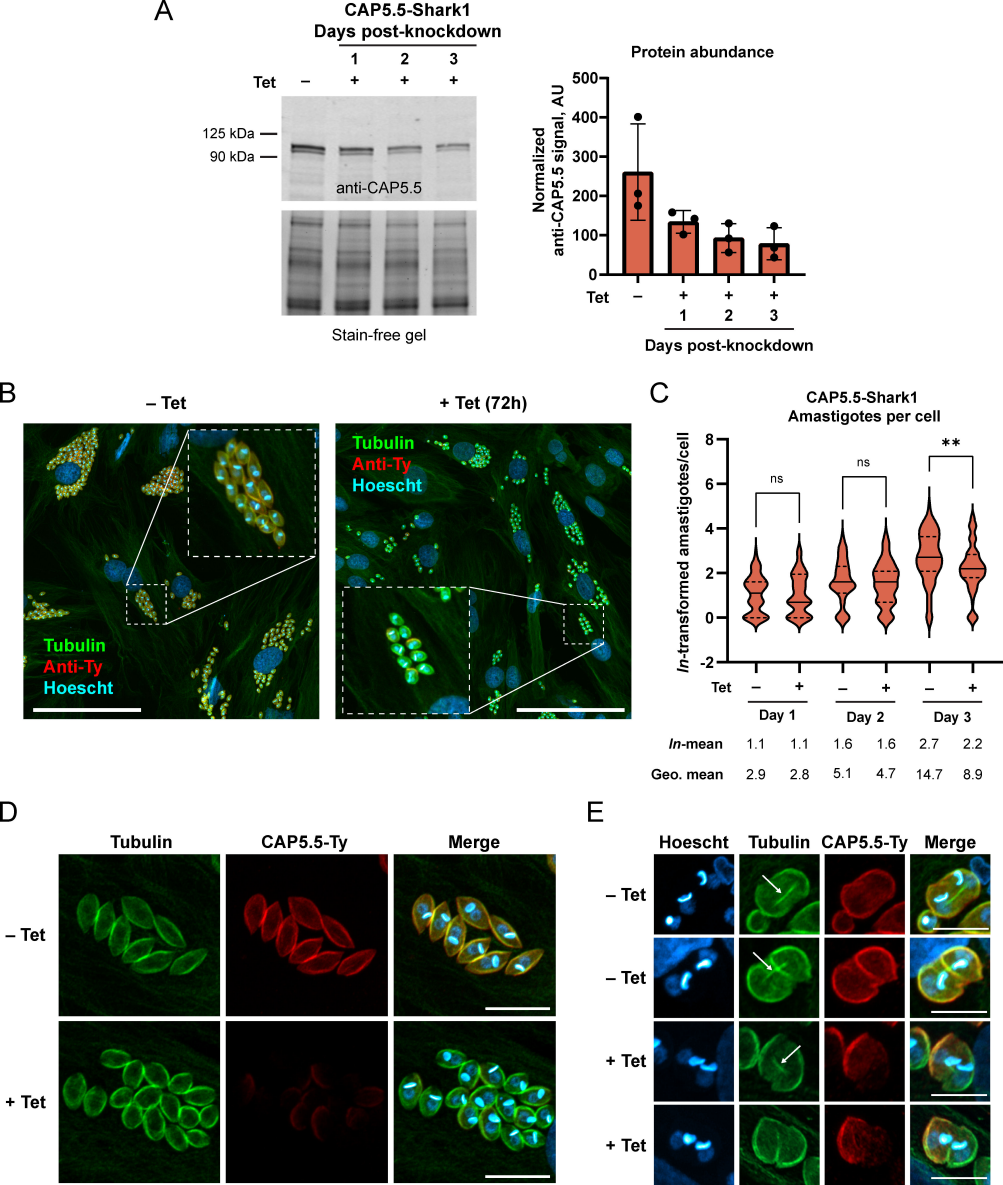
Effect of CAP5.5 knockdown on amastigote proliferation and kinetoplast and nucleus configuration. (**A**) Left panel: Representative western blot of CAP5.5 abundance from a knockdown time course. The membrane was stained with anti-CAP5.5 3D8 antibody. Stain-free gel shows total protein loading before transfer. Right panel: Quantification of 3D8 signal after tetracycline (Tet) addition at the indicated time points from three independent replicates. Error bars represent std. dev. (**B**) Amastigote proliferation was determined using immunofluorescence microscopy by counting the number of amastigotes per cell in infected cardiomyocyte monolayers stained with Hoescht 33342 (DNA), TAT1 antibody (tubulin), and anti-Ty antibody (CAP5.5) before (left panel) and after (right panel) Tet addition. Scale bars = 100 µm. (**C**) Amastigotes per cell at the indicated time points. At least 24 infected cardiomyocytes were analyzed at each time point in three independent replicates. The dataset was transformed using the natural log (ln) of each number to correct for strong right-skewness and stabilize variance for statistical analysis. An ANOVA with Holm-Sidak’s multiple comparisons test was performed on the ln-transformed dataset to assess statistical significance between time points. For – Tet vs 24 h, *P* = 0.9971. For – Tet vs 48 h, *P* = 0.9503. For – Tet vs 72 h, *P* = 6.4 x 10^−3^. Below the graph, the counts of amastigotes per cell at each time point are reported as geometric means of the untransformed values and as means of the ln-transformed values. **P ≤ 0.01. (**D**) Immunofluorescence microscopy of CAP5.5-Shark1 amastigotes before (top row) and after (bottom row) 72 h Tet treatment. Trypanosomes were stained with Hoescht 33342, TAT1 antibody, and anti-Ty antibody. Scale bars = 10 µm. (**E**) Immunofluorescence microscopy of post-mitotic intracellular CAP5.5-Shark1 amastigotes before (top two rows) or after (bottom two rows) 72 h Tet treatment. Arrows indicate mitotic spindle (if present). Scale bars = 10 µm; ns, not significant.

CAP5.5 knockdown produces similar defects when comparing insect-stage *T. cruzi* (epimastigotes) and insect-stage *T. brucei* (procyclics) (20). However, the mammalian stages differ significantly between the two trypanosome species. *T. cruzi* infects host cells and proliferates intracellularly as amastigotes, which are relatively immobile, while *T. brucei* proliferates as highly motile, extracellular, bloodstream-form trypomastigotes. Importantly, CAP5.5 has not yet been studied in trypanosomatid life-cycle stages with reduced motility. To functionally characterize CAP5.5 in amastigote-stage *T. cruzi*, we studied the effects of knockdown in trypanosomes collected from infected host cells. First, we confirmed CAP5.5 knockdown in a 3-day time course by western blotting (Fig. 5A, left panel). After 3 days post-Tet addition, the average CAP5.5 signal decreased by 69.7% (Fig. 5A, right panel). Curiously, at the same time point (72 h), Tet treatment of Y-strain (parental line) amastigotes produced the opposite effect, increasing the average CAP5.5 signal by nearly 50% (Fig. S3A). Having confirmed knockdown, we then tested whether CAP5.5 was required for proliferation by counting the number of amastigotes per infected host cell (Fig. 5B). CAP5.5 knockdown had a mild effect on amastigote proliferation, decreasing the average number of amastigotes per cell (geometric mean) by 39.5% after 3 days (Fig. 5C) (*P* = 6.4 × 10^−3^, Holm-Sidak’s performed following ANOVA on *ln*-transformed data set). Tet treatment had no effect on Y-strain amastigote proliferation at this timepoint (*P* = 0.14, Holm-Sidak’s performed following ANOVA on *ln*-transformed data set) (Fig. S3B). We conclude that CAP5.5 is required for optimal proliferation in amastigotes.

**Fig 6:**
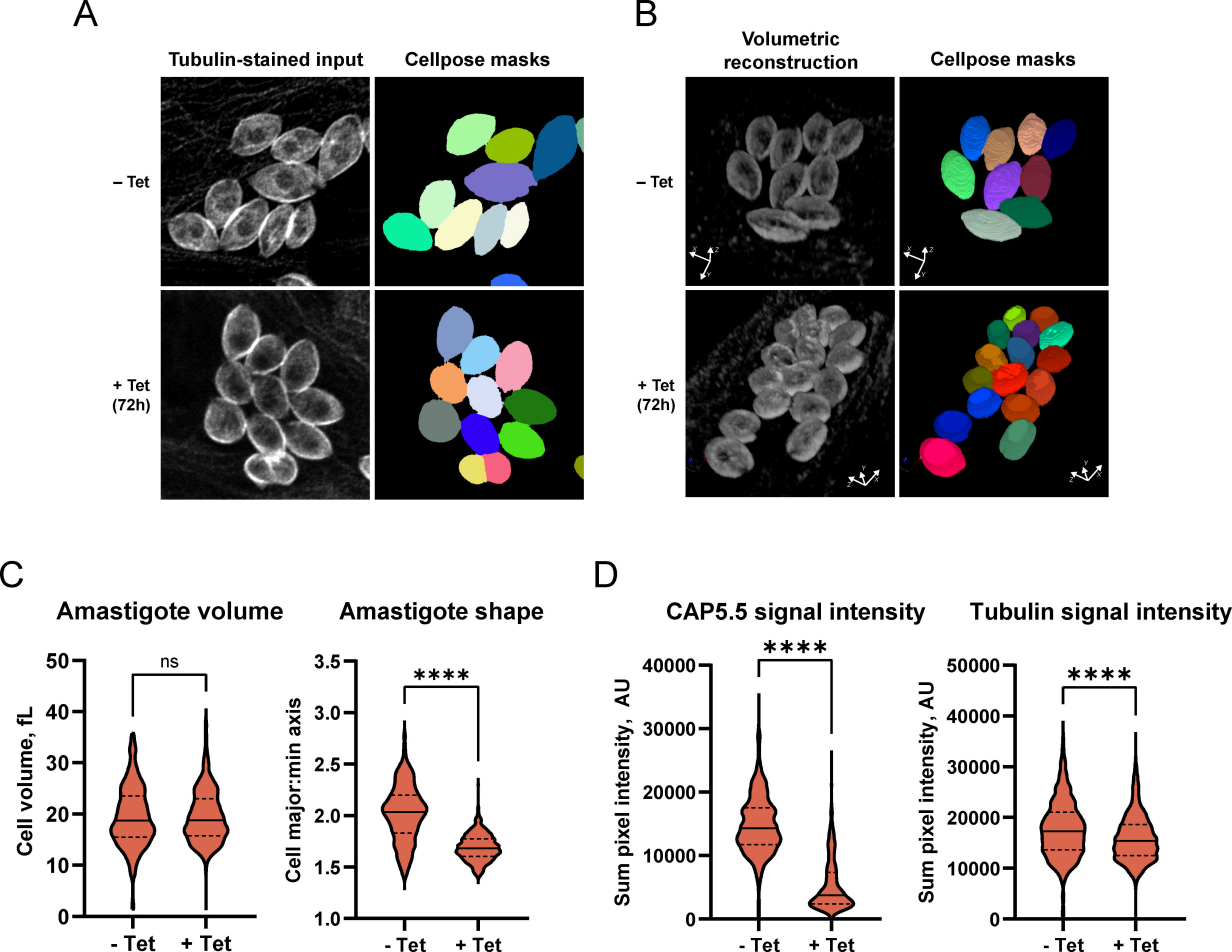
Semi-automated image masking and volumetric characterization of amastigotes after CAP5.5 knockdown. (**A**) Maximum intensity projections of Z-stack images of intracellular CAP5.5-Shark1 amastigotes stained with TAT1 antibody (tubulin) before (top row) or after (bottom row) tetracycline (Tet) addition. Images were segmented and cell masks created using Cellpose-SAM. (**B**) Volumetric reconstruction of Z-stack images of TAT1-stained amastigotes before (top row) or after (bottom row) Tet addition. XYZ arrows in the corners of each image indicate three-dimensional orientation relative to the original orientation of the acquired image. (**C–D**) Measurements of 3D cell masks before and after 72 h CAP5.5 knockdown. *N* > 650 cells for each sample. Student’s T-test was used to test for statistical significance between measurements. (**C**) Left panel: Amastigote volume (*P* = 0.58). Right panel: Shape measurement (Major:Minor axis ratio) (*P* < 0.0001). (**D**) Left panel: Anti-Ty signal intensity (*P* < 0.0001). Right panel: TAT1 signal intensity (*P* < 0.0001). ns, not significant. **** P < 0.0001.

In epimastigotes, CAP5.5 knockdown inhibited organelle segregation (Fig. 3B) and produced defects in cytokinesis (Fig. 3C). In amastigotes, however, we did not observe any obvious defects in the number of kinetoplasts or nuclei after 72 h of knockdown (Fig. 5D and Fig. S3C). An IFA with anti-Ty and anti-tubulin (TAT1) antibodies revealed a distinctive pattern of CAP5.5 signal loss (Fig. 5E), where CAP5.5 was detectable on one side of a cell but not on the other. This was particularly apparent in dividing amastigotes, where CAP5.5 was detectable in the mother cell but not in the daughter cell (Fig. 5E). Unlike in epimastigotes, no apparent defects in cytokinesis were detected (Fig. S3C), suggesting that CAP5.5 does not play an essential role in cytokinesis in amastigotes. CAP5.5 knockdown nonetheless appeared to alter both the size and shape of amastigotes stained with anti-tubulin antibody (Fig. 5D). To test whether these changes were statistically significant, we obtained 3D masks of amastigotes (Fig. 6A and 6B) from z-stack images of amastigote nests using Cellpose-SAM (26) and CellProfiler (27) as we did with epimastigotes (Fig. 4). Then, we measured the volume and roundness of the masks (Fig. 6C), and the intensity of anti-Ty and anti-tubulin signal in the parasite masks (Fig. 6D). Following 72 h knockdown, the average amastigote volume did not change (– Tet = 19.4 fl, +Tet = 19.6 fl) (*P* = 0.58, Student’s T-test), but the average Major:Minor axis ratio decreased by 17.4%, from 2.0 to 1.7 (Fig. 6) (*P* < 0.0001, Student’s T-test). Finally, we confirmed the efficacy and specificity of CAP5.5 knockdown by measuring anti-Ty and anti-tubulin signal intensity. CAP5.5-Ty signal (geometric mean) decreased 70.4% from 13,923 AU to 4,128 AU (*P* < 0.0001, Mann–Whitney), while the average tubulin signal remained largely unchanged, decreasing 8.1% (*P* < 0.0001, Mann–Whitney) (Fig. 6D). We conclude that CAP5.5 is not essential for maintaining cell volume but does contribute to the maintenance of cell shape.

### Simultaneous knockdown and rescue expression of CAP5.5 in epimastigotes

**Fig 7:**
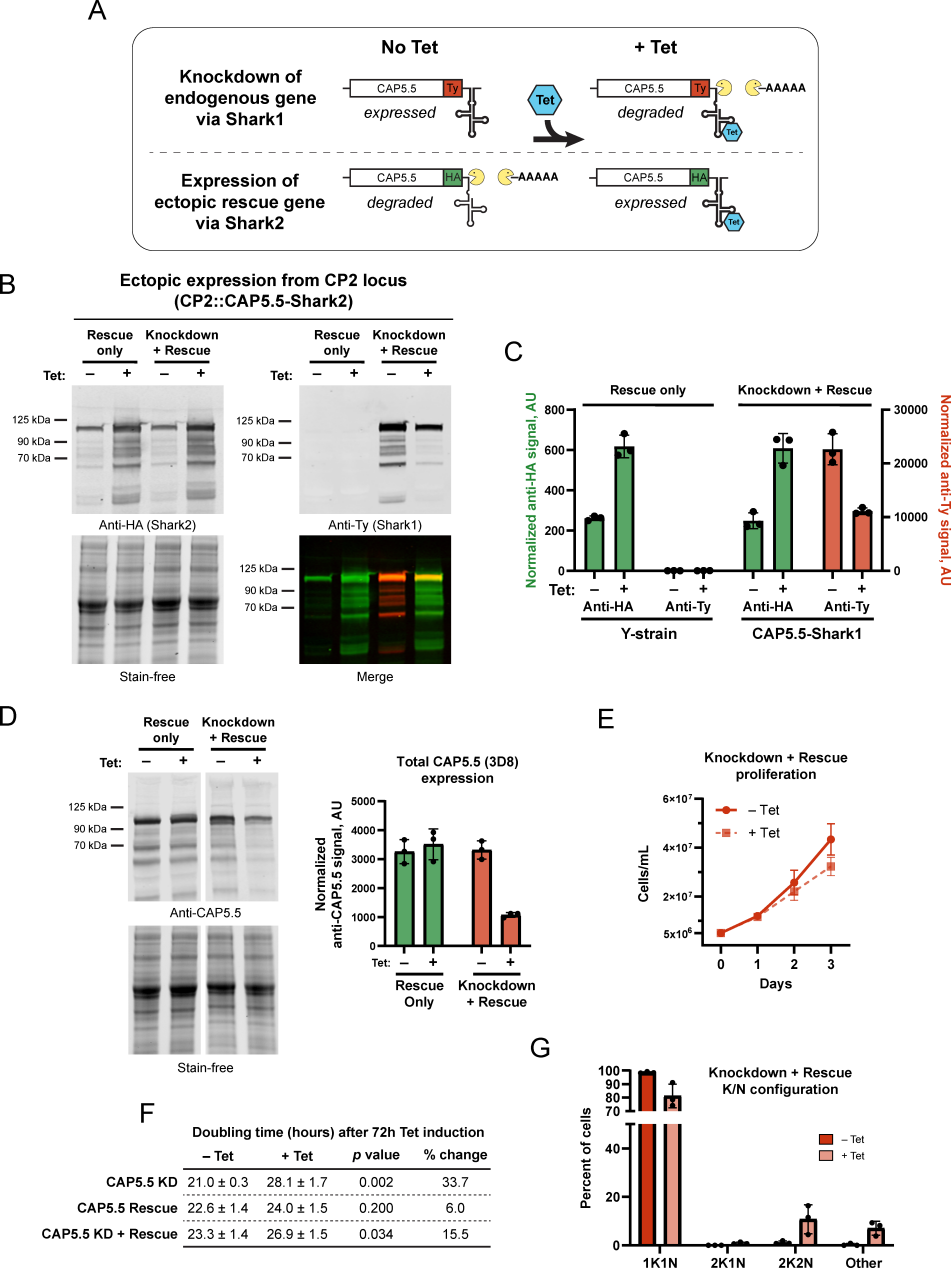
Simultaneous knockdown of endogenous CAP5.5 and expression of ectopic CAP5.5. (**A**) Cartoon illustration of the dual aptazyme concept, which allows silencing of endogenous CAP5.5-Ty and expression of ectopic CAP5.5-HA. (**B**) Representative western blot of CAP5.5-HA (top left) and CAP5.5-Ty (top right) abundance with or without 72 h tetracycline (Tet) treatment. Stain-free gel (lower left) illustrates total protein loading before transfer. In the merge (bottom right), the anti-HA signal is green, and the anti-Ty signal is red. (**C**) Quantification of anti-HA and anti-TY signal from western blots from three independent experiments. Error bars indicate std. dev. **D**. Left panel: Representative western blot of total CAP5.5 abundance (labeled with anti-CAP5.5 antibody 3D8) in “Rescue only” and “Knockdown + Rescue” cells before and after 72 h Tet treatment. Right panel: quantification of anti-CAP5.5 signal from western blots from three independent experiments. Error bars represent std. dev. (**E**) Proliferation of “Knockdown + Rescue” epimastigotes with or without Tet treatment from three independent experiments. Error bars represent std. dev. (**F**) Average doubling times (± std. dev) for 0–72 h calculated from data presented in E and in Fig. 2B (for “CAP5.5 KD” sample). A Student’s T-test was used to test for statistical significance between doubling times. The percent change in average doubling time after Tet treatment is presented. (**G**) Quantification of the number of kinetoplasts and nuclei per cell at the indicated time points after Tet addition. The average percentage of each cell type from three independent replicates is presented. Error bars represent std. dev. *N* > 150 trypanosomes at each time point.

The SHARK system is comprised of three types of hammerhead-based aptazymes: one where Tet activates ribozyme cleavage (Shark1), one where Tet inactivates ribozyme cleavage (Shark2), and one where theophylline activates ribozyme cleavage (Shark3) (8). We hypothesized that by using both Shark1 and Shark2 ribozymes in the same cell line, we could knock down endogenous CAP5.5 via Shark1 and simultaneously induce expression of an ectopic “rescue” copy of CAP5.5 via Shark2 (Fig. 7A schematic). To do this, we PCR-amplified the CAP5.5 locus from *T. cruzi* genomic DNA (gDNA) and inserted it into a vector containing a Shark2 sequence, 3× HA tag, GAPDH UTR sequence, and a puromycin resistance cassette. We then PCR amplified this combined Shark2-tagged CAP5.5 cassette and drug marker and used it to replace a gene that we have previously determined to be non-essential for proliferation (data not shown), TcYC6_0072560 (cytostome protein 2 [CP2]) (Fig. S4A) (29). We generated CP2::CAP5.5-Shark2 epimastigote knock-ins in two distinct lineages: first, the CAP5.5-Shark1 line used in this study, and second, in Y-strain (parental line) trypanosomes. We refer to CP2::CAP5.5-Shark2 cells in the CAP5.5-Shark1 background as a “Knockdown + Rescue” line, and we refer to CP2::CAP5.5-Shark2 cells in the Y-strain background as “Rescue Only” cells. After transfecting, we selected trypanosome clones from both lineages where both loci of CP2 had been replaced with CAP5.5-Shark2, as determined by diagnostic PCR (Fig. S4B).

To see whether both Shark1 and Shark2 ribozymes were active, we treated epimastigotes with Tet for 72 h and then checked for knockdown of CAP5.5-Ty and expression of CAP5.5-HA by western blot (Fig. 7B). In “Rescue Only” cells, Tet addition induced CAP5.5-HA expression, increasing the average HA signal by 135% (Fig. 7C). In “Knockdown + Rescue” cells, Tet addition decreased average CAP5.5-Ty signal by 51%, while CAP5.5-HA signal increased by 145% (Fig. 7C). Although Tet addition did promote expression of “rescue” CAP5.5-HA, the average total CAP5.5 abundance only increased by 7.3% (Fig. 7D, “Rescue Only”) leading to an inability to fully complement the knockdown of endogenous CAP5.5 (Fig. 7D, “Knockdown + Rescue”). Finally, we examined the effect of CAP5.5 knockdown and rescue on epimastigote proliferation (Fig. 7E and quantified in 7F) and cell cycle progression (Fig. 7G). After 72 h, in “Knockdown and Rescue” cells, the average proliferation rate decreased by 15%, while in “Knockdown Only” cells, the average rate decreased by 33.7% (Fig. 7F). In “Knockdown + Rescue” cells, post-mitotic cells (e.g., those having at least two nuclei) comprised 18.0% of the population (Fig. 7F), compared to “Knockdown Only” cells, where post-mitotic cells comprised 55.9% of the population after 72 h (Fig. 3). Finally, in “Rescue Only” cells, we confirmed that the expression of CAP5.5 had no effect on proliferation or kinetoplast/nucleus configuration (Fig. S4C and D, respectively). These data demonstrate that Shark1 and Shark2 ribozymes can be used simultaneously to knock down expression of an endogenous gene and induce expression of an ectopic copy.

## DISCUSSION

Over the past decade, new tools for studying *T. cruzi* have transformed the field. The introduction of CRISPR/Cas9 (6, 7) in 2014 was an inflection point for genetic studies and represented the most important advancement since the sequencing of the first *T. cruzi* genome in 2005 (30, 31). CRISPR/Cas9 greatly improved transfection efficiency and finally made basic genetic manipulation a feasible endeavor (31). In 2021, developments in long-read sequencing and proximity ligation mapping were leveraged to significantly improve the quality of genome assemblies for two primary lab strains of *T. cruzi* (5). Most recently, in 2025, our group developed the first conditional gene knockdown system (SHARK) for *T. cruzi* (8) and has since applied it to dozens of genes, and it has been adopted and used successfully by another research group (32). Together, these advances now enable in-depth molecular studies of gene function in *T. cruzi* that previously were only possible in *Trypanosoma brucei*.

Here, using CAP5.5 as a model, we have (i) demonstrated the ability of the SHARK system to simplify, for the first time, the validation of antibodies raised against putative essential genes (Fig. 1), (ii) introduced the use of semi-automated image segmentation to enable high-resolution volumetric measurements of trypanosomes (Fig. 4 and Fig. 6), and (iii) shown that endogenous gene knockdown and ectopic gene expression can be simultaneously controlled with a single ligand (Fig. 7). Functionally, we show that CAP5.5 is necessary for proliferation and cytokinesis in epimastigotes (Fig. 3), whereas its depletion in amastigotes leads to a less severe phenotype, indicating a distinct life-cycle stage dependence on this array-associated factor.

In epimastigotes, CAP5.5 knockdown leads to failed cytokinesis (Fig. 3C) and pronounced changes to cell shape and volume (Fig. 4). Such phenotypes are often observed following knockdown of microtubule array-associated proteins in trypanosomatids. Previous work on CAP5.5 in *T. brucei* provides a useful point of comparison for our findings in *T. cruzi*. TbCAP5.5 was originally identified as a life-cycle-regulated, calpain-related protein that is tightly associated with the subpellicular cytoskeleton, where dual N-myristoylation and S-palmitoylation are thought to promote stable membrane-cytoskeleton attachment (17, 33). Genetic studies in *T. brucei* demonstrated that CAP5.5 and its bloodstream-stage paralog, CAP5.5V, are each essential for normal cell morphogenesis and cytokinesis, with RNAi depletion causing cell rounding, kinetoplast-nucleus mispositioning, and cleavage furrow defects that closely resemble the phenotypes we observe in *T. cruzi* epimastigotes (18). More recently, BioID using TbCAP5.5 as bait identified an interacting cohort of cytoskeleton-associated proteins, including CAP50, CAP52, and CAP42, whose depletion likewise disrupts subpellicular array organization and cell division, supporting the idea that CAP5.5 functions as part of a larger structural and regulatory module (28). Mispositioning of the nucleus and kinetoplast and severe cytokinesis defects were also observed in *T. brucei* following the knockdown of the array-associated protein TbAIR9 as well (34). Since similar phenotypes are reported for different microtubule array components, this raises the possibility that these proteins may function in intimate complexes with each other. Many cytoskeletal-associated proteins are thought to contribute to the remodeling of the array and the stability of microtubules (11); thus, their knockdown may alter array stability. In support of this, destabilization of microtubules with the drug oryzalin in *T. cruzi* epimastigotes mimics some elements of array protein knockdown by inducing cell rounding (12), as seen with CAP5.5 knockdown (Fig. 4). Our data in *T. cruzi* thus extend this model by showing that CAP5.5 appears to be specifically required in the highly motile parasite stage, where mechanical demands on the cytoskeleton are greatest, and by revealing polarized turnover dynamics after knockdown, suggesting that CAP5.5 turnover is highest at sites of ongoing array remodeling. Together, these data suggest that in *T. cruzi,* CAP5.5 also likely functions as an integral member of a complex of interdependent proteins that together promote microtubule stability and array integrity.

The pronounced contrast in CAP5.5 knockdown phenotypes between epimastigotes (Fig. 3) and amastigotes (Fig. 5) suggests that distinct life-cycle stages impose different demands on the microtubule array, as proposed by Sinclair and deGraffenried (11). While we cannot strictly rule out the possibility that the observed phenotypic differences result from the slightly lower knockdown efficiency in amastigotes, the near-total absence of cytokinesis defects suggests a genuine biological divergence in CAP5.5 requirement. A major distinction between epimastigotes and amastigotes is found when comparing motility. Epimastigotes are highly motile with a rapidly beating flagellum that extends past the cell body (35), while intracellular amastigotes are relatively immobile and possess a much shorter flagellum that does not appear able to support productive parasite motility (36, 37). Flagellar beating in epimastigotes creates undulations along a portion of the cell body (35), necessitating flexibility and resilience in the microtubule array (11). Although microtubules are inherently resilient to mechanical forces, repeated bending can “fatigue” and weaken them over time (38), implying that the array must be continually maintained or repaired to withstand ongoing mechanical stresses. The observation that CAP5.5 knockdown produces dramatic morphological and cytokinetic defects in motile forms but not in the relatively immobile amastigote form is consistent with a role for CAP5.5 in repairing or reinforcing microtubules that experience motility-associated damage. To achieve this, CAP5.5 may modulate interactions between microtubule-modifying enzymes (such as acetylases [12], kinases [39], and tyrosine ligases [40]) and the array. The acetylation of α-tubulin has been linked to microtubule stability (12) and flexibility (41) and has been proposed as a protective mechanism against motility-associated array damage (11). Interestingly, treatment of *T. cruzi* epimastigotes with a deacetylase inhibitor interfered with basal body segregation (42), and overexpression of the *T. cruzi* α-tubulin acetyltransferase inhibited proliferation and induced epimastigote rounding (see Fig. 5 in reference 12). These data support the existence of a link between tubulin acetylation, organelle positioning, and potential motility-associated damage.

After induction of Shark1-mediated gene knockdown, CAP5.5-Ty is depleted from the microtubule array in a linear manner (Fig. 3) along the longitudinal axis of the cell body. Because aptazymes silence genes by degrading mRNA transcripts and preventing protein synthesis, depletion of existing protein requires proteasome-mediated turnover or division-mediated dilution. The rate of CAP5.5 protein turnover appears to differ throughout subdomains (43) of the microtubule array. CAP5.5 turnover is more rapid at the posterior and dorsal regions of the cell, where microtubule polymerization and array remodeling occur (13, 44). In *T. brucei*, another array-associated protein, TbWCB, co-localizes with TbCAP5.5 throughout the cell body and is likewise linearly depleted along the length of the cell body after RNAi knockdown (45). These data suggest that protein turnover of microtubule-array-associated proteins in kinetoplastids is most rapid where cytoskeletal polymerization and array remodeling occur (20).

In this work, we presented a proof of concept that our conditional knockdown system is useful for the validation of antibody specificity and that different aptazymes (such as Shark1 and Shark2) can be combined in a single-cell line to coordinate endogenous knockdown and ectopic expression of a rescue copy with a single ligand (Fig. 7A). Tet treatment activated knockdown of endogenous CAP5.5 via Shark1 and expression of ectopic CAP5.5 via Shark2 in “Knockdown + Rescue” (K + R) cells (Fig. 7B). Ectopic CAP5.5 expression was easily detected with an anti-HA antibody (Fig. 7B), but the contribution of “Rescue” CAP5.5-HA to the total intracellular pool of CAP5.5 was minimal (Fig. 7D). Despite minimal expression of “Rescue” CAP5.5-HA, this was still sufficient to partially rescue the proliferation defect (Fig. 7E and 7F) and cytokinesis phenotype (Fig. 7G) associated with CAP5.5 knockdown. Our preliminary studies revealed that episomal expression of “Rescue” CAP5.5-HA (under control of a *T. cruzi* ribosomal promoter [46]) in the pMiniTrex (47) vector induced high-level overexpression, leading us to place “Rescue” CAP5.5-HA in the CP2 locus to better control expression levels. Future identification of a genomic locus or 3′ UTR sequence supporting fully tunable expression of transgenes could significantly improve genetic studies in *T. cruzi*. We anticipate that further development of our dual aptazyme knockdown/expression system could allow for conditional mutagenesis studies of target proteins. For example, an active enzyme could be knocked down with Shark1, and a catalytically inactive version could be simultaneously expressed using Shark2 with a single ligand.

In summary, our findings extend the role of CAP5.5 in trypanosomatid biology by implicating this protein in the maintenance of the subpellicular microtubule array under motility-associated mechanical stress and highlighting stage-specific requirements for array-associated factors. The methodological advances we describe, including SHARK-based conditional knockdown, dual-aptazyme knockdown/rescue, and semi-automated volumetric analysis using open-source software, provide generalizable tools and workflows that can be readily employed by others. These resources should facilitate systematic dissection of basic *T. cruzi* biology, bringing studies of this pathogen closer to the level of mechanistic detail long available in *T. brucei*.

## MATERIALS AND METHODS

### Trypanosome culture

Y-strain trypanosomes were maintained as epimastigotes at 28°C in liver infusion tryptose (LIT) medium with 15% heat-inactivated (HI) FBS as described previously (8). Metacyclic trypomastigotes were obtained by prolonged incubation (>10 days) of epimastigotes in LIT, then treating cultures with normal human serum (NHS) to lyse epimastigotes. NHS-treated trypanosome cultures were added to H9C2 rat cardiomyocytes (maintained in DMEM [1.5 g/L bicarbonate] with 10% HI FBS at 37°C with 5% CO_2_) in monolayers to infect them and form amastigote nests. Monolayers were washed, and after several days, bloodstream form trypomastigotes were released into the culture medium. These tissue culture-derived trypomastigotes (TCTs) were collected and used for subsequent re-infection of monolayers for infection assays or continued propagation.

### Expression and purification of recombinant CAP5.5 in *E. coli*

The *Trypanosoma cruzi* CAP5.5 gene (TcYC6_0107360) encoding the putative cytoskeleton-associated protein was PCR amplified from Y-strain gDNA and cloned into pQE-80 for 6xHis tagging using Gibson assembly. A sequence-verified plasmid was introduced into BL21 (DE3) for protein expression. A single colony was grown overnight in LB with carbenicillin (100 µg/mL), diluted 1:100, and CAP5.5-6xHis expression was induced with 0.4 mM IPTG at OD₆₀₀ ≈ 0.6. Expression was performed overnight at 16°C to enhance solubility. Cells were harvested (5,000 × *g*, 15 min, 4°C), resuspended in lysis buffer (50 mM Tris-HCl pH 8.0, 300 mM NaCl, 10 mM imidazole, 5% glycerol, 1 mM DTT, and protease inhibitors), and lysed twice using a French press at 15,000–18,000 psi. Lysates were clarified (45,000 × *g*, 60 min, 4°C) and filtered (0.45 µm). The supernatant was applied to a Ni²^+^–NTA HisTrap column (Cytiva) pre-equilibrated with the same buffer. After washing with 20–40 mM imidazole, the bound protein was eluted using a step gradient of 100–300 mM imidazole in equilibration buffer. Fractions containing CAP5.5 (~90 kDa) were pooled, desalted into PBS (pH 7.4) using PD-10 columns, and concentrated with Amicon Ultra-15 (30 kDa MWCO) centrifugal filters (Millipore Amicon) to ~ 1 mg/mL. Protein purity and identity were confirmed by Stain-free SDS-PAGE and anti-His Western blot. The purified, concentrated CAP5.5 was aliquoted and stored at −80°C for subsequent monoclonal antibody production.

### Anti-CAP5.5 antibody production

Monoclonal antibodies against *Trypanosoma cruzi* CAP5.5 were produced under contract by the Bioexpression and Fermentation Facility (BFF) at the University of Georgia (Athens, GA). All animal protocols were approved by the University of Georgia Institutional Animal Care and Use Committee (IACUC).

*Immunization*: Female BALB/c mice (5 weeks to 1-year old) were immunized with purified recombinant *T. cruzi* CAP5.5 protein. The initial immunization consisted of a subcutaneous injection of 100 µL of antigen emulsified in Complete Freund’s Adjuvant (CFA). Subsequent booster injections were administered intraperitoneally (IP) with antigen in Incomplete Freund’s Adjuvant (IFA) at 21-day intervals. Antibody titers were monitored via test bleeds collected from the tail vein or saphenous vein. The animal exhibiting the highest titer was selected for fusion and received a final pre-fusion boost of 40–50 µg of antigen in phosphate-buffered saline (PBS) via IP injection 3 days prior to spleen harvest.

*Cell fusion and hybridoma selection*: Splenocytes were harvested and fused with SP2/0 murine myeloma cells using polyethylene glycol (PEG) 1000 at a ratio of 5:1 (spleen:myeloma). Fused cells were resuspended in Iscove’s Modified Dulbecco’s Medium (IMDM) supplemented with 20% fetal bovine serum (FBS) and distributed into 96-well plates. Hybridomas were selected using Hypoxanthine-Aminopterin-Thymidine (HAT) medium.

*Screening and isotyping*: Hybridoma supernatants were screened for antibody production by indirect enzyme-linked immunosorbent assay (ELISA). Plates were coated with the recombinant CAP5.5 antigen in borate saline buffer (pH 8.5) and blocked with 1% bovine serum albumin (BSA) in PBS. Bound antibodies were detected using species-specific secondary antibodies conjugated to alkaline phosphatase or horseradish peroxidase. Positive clones were expanded and isotyped by ELISA to determine heavy and light chain classes.

### CRISPR/Cas9-mediated endogenous tagging with SHARK tag

A Shark1-3xTy tagging cassette was PCR amplified with primers containing overhangs targeting the cassette to the C-terminus of the CAP5.5 CDS. A guide RNA directing Cas9 (7) to the CAP5.5 locus was inserted by site-directed mutagenesis as described in reference 8. Transfection and selection of homozygous-tagged clones were performed as described previously (8).

### Trypanosome proliferation assays

#### Epimastigotes

Epimastigote cultures were prepared at a density of 2.5 × 10^6^/mL in LIT and then split into two flasks. Tet was added to a final concentration of 5 µg/mL to one flask. Both flasks were incubated at 28°C, and the cell density was determined by a coulter counter every 24 h for 5 days, and then every 48 h for the next 4 days. Cultures were diluted periodically with fresh pre-warmed LIT ± 5 µg/mL Tet to maintain log-phase growth. Epimastigote doubling times were calculated as before (8). The statistical significance of changes to doubling times was calculated as previously described.

#### Amastigotes

Tissue culture-derived trypomastigotes (10^6^) were mixed with 5 × 10^4^ trypsinized H9C2 cells and added to wells from a 24-well plate containing glass cover slips at the bottom of the wells. Plates were incubated at 37°C × 24 h, then the media was removed, and cells were washed 2× with pre-warmed HBSS (with Ca^2+^ and Mg^2+^). Pre-warmed DMEM (1 mL) (±5 µg/mL Tet) was added to each well, and plates were incubated at 28°C for 24, 48, or 72 h. At the indicated time points, samples were collected and processed for immunofluorescence as before (8). Image grids (5 × 5) were acquired using a Zeiss Axio Observer microscope equipped with a 63× objective and a Yokogawa CSU-W1 SoRa super resolution confocal scanning unit, and the number of amastigotes per infected host cell was determined. Gamma correction (0.7) was applied to the Hoescht channel to aid in the visualization of amastigotes. Since the data set of counts of amastigotes per cell was strongly right-skewed, the counts were log transformed (*ln*) to stabilize variance. The statistical significance of the difference between log-transformed mean amastigotes per cell at each time point was compared using a one-way ANOVA with a post hoc Holm-Sidak’s multiple comparisons test.

### SDS-PAGE and Western blot

Trypanosomes were harvested and processed for Western blots as previously described (8). Approximately 5 × 10^6^ epimastigote and 2.5 × 10^6^ amastigote cell equivalents were loaded per lane of a Stain-free (Bio-Rad). Protein transfer, antibody incubation, membrane image acquisition, and densitometry were performed as previously described (8). For this work, Anti-Ty (BB2) was used at a 1:10,000 dilution, anti-HA (clone 6E2, Cell Signaling Technology) was used at a 1:10,000 dilution, and anti-CAP5.5 3D8 antibody was used at a 1:1,000 dilution.

### Acyl PEGyl exchange gel shift assay

The Acyl-PEGyl Exchange Gel Shift (APEGS) assay was performed as described in reference 23. This method detects protein palmitoylation by attaching a 10 kDa PEG molecule to cysteine residues that were previously palmitoylated, resulting in a detectable mass shift by Western blot. Briefly, parasites were lysed in a buffer containing 4% SDS, and the disulfide bonds in the protein lysate were reduced with TCEP at 55°C for 1 h. All free cysteine residues were subsequently blocked by an overnight incubation with N-ethylmaleimide (NEM) at room temperature. Following protein precipitation with methanol/chloroform, the palmitoyl-thioester linkages were cleaved by incubating the samples in 2 M hydroxylamine for 1 h at 37°C. After a second precipitation, the newly exposed cysteine residues were labeled by a 2-h incubation with 7 mM mPEG-MAL-10k, with a parallel sample incubated without mPEG-MAL-10k serving as a negative control. A final precipitation was performed to remove excess mPEG, and the protein pellet was resuspended in Laemmli buffer for analysis by SDS-PAGE and Western blot.

### Membrane staining of epimastigotes and fluorescence microscopy

10^6^ epimastigotes from log-phase cultures were collected, pelleted at 1,000 × *g* for 3 min, and washed 1× with 250 µL ice-cold HBSS. After washing, cells were pelleted again at 1,000 × *g* for 3 min at 4°C and resuspended in 50 µL ice-cold HBSS, and 1 µL 50 µM mCLING (24) was added, and cells were mixed by pipetting. Fifty microliters of 2× fixative (8% PFA + 0.5% glutaraldehyde) was added, and cells were mixed by pipetting. Approximately 2 × 10^5^ epimastigotes (20 µL) were transferred onto a poly-L-lysine-coated glass coverslip and allowed to settle onto the coverslip and adhere for 1 h in a humidified chamber in the dark. The coverslip was then washed 2× with 500 µL PBS in a well of a 24-well plate. The coverslip was then carefully removed from the well and allowed to air-dry before being mounted on a glass slide with 2 µL of Prolong Gold mounting medium containing 5 µg/mL of Hoescht 33342. For kinetoplast/nucleus quantification and volumetric measurements after CAP5.5 knockdown, z-stack images were acquired on a Nikon Eclipse Ti2E widefield microscope equipped with a 100× objective and deconvolved using Ideas software (Nikon). Images were processed using Fiji (48). For kinetoplast/nucleus quantification after simultaneous CAP5.5 knockdown and rescue expression, z-stack montages were captured using a Zeiss Axio Observer microscope equipped with a 63× objective and a Yokogawa CSU-W1 SoRa super resolution confocal scanning unit. Montage images were stitched together using Slidebook software, and images were then processed using Fiji.

### Immunofluorescence microscopy

#### Epimastigotes

Epimastigotes were harvested and adhered to a glass coverslip as described above. After adhering cells, coverslips were placed into the wells of a 24-well plate and incubated with 0.1% Triton X-100 in PBS for 10 min to permeabilize trypanosomes. Blocking was performed with 1% BSA in PBS for 1 h. Antibody labeling of trypanosome alpha tubulin and CAP5.5-Ty was performed with 1:1,000 mouse TAT1 antibody and 1:1,000 rabbit anti-Ty antibody, respectively, in 1% BSA in PBS. Secondary antibody labeling was performed with 1:1,000 Alexa Fluor 488 goat anti-mouse antibody and 1:1,000 Alexa Fluor 594 goat anti-rabbit antibody. Images were acquired on a Zeiss LSM980 Airyscan2 confocal microscope equipped with a 100× objective or on a Zeiss Axio Observer microscope equipped with a 63× objective and a Yokogawa CSU-W1 SoRa super resolution confocal scanning unit with the 4× Magchanger activated.

#### Amastigotes

Infected host cell monolayers grown on glass coverslips were processed as described previously (8). For kinetoplast/nucleus quantification and volumetric measurements after CAP5.5 knockdown, images were acquired on a Zeiss LSM980 Airyscan2 confocal microscope equipped with a 100× objective. For the amastigote proliferation assay, z-stack montages were captured using a Zeiss Axio Observer microscope equipped with a 63× objective and a Yokogawa CSU-W1 SoRa super resolution confocal scanning unit. Montage images were stitched together using Slidebook software, and images were then processed using Fiji.

### Semi-automated cell masking and volumetric measurements

Z-stack images of epimastigotes stained with mCLING or amastigotes stained with TAT1 antibody were used to train custom image segmentation models in Cellpose-SAM (v 4.0.6) (26) in XY, ZY, and ZX dimensions. Single-channel z-stack images were prepared for processing in Fiji by adjusting brightness and contrast and then batch processed in Cellpose-SAM using the appropriate (e.g., epimastigote or amastigote) custom model. Masks were exported from Cellpose-SAM and imported into CellProfiler (v 4.2.8), where they were measured and overlaid onto raw z-stack images to measure antibody signal overlapping with individual cell masks. Measurements were exported from CellProfiler and graphed using GraphPad Prism 10.6.1. Three-dimensional reconstructions were created using Icy (49). For a detailed tutorial, see the attached supplemental file.

### CAP5.5 sequence alignment and domain annotation

Amino acid sequences of CAP5.5V (Tb927.8.8330), CAP5.5 (Tb927.4.3950), and the *T. cruzi* ortholog (TcYC6_0107360) were retrieved from TriTrypDB. Multiple sequence alignment was performed using MAFFT (v7.526) using default parameters with L-INS-i refinement. Pairwise amino acid identity was calculated from the aligned sequences excluding gap positions, yielding 78.9% identity between the two *T. brucei* paralogs, and 46.2% and 47.2% identity between each *T. brucei* paralog and the *T. cruzi* ortholog, respectively. Functional domains and motifs were identified through comparison with previously characterized calpain-related proteins in kinetoplastids (15). The N-terminal dual acylation motif (myristoylation and palmitoylation signals), catalytic triad positions (17), proline-rich region, central calpain-like domain, and acidic C-terminal tail were delineated based on published sequence features and domain boundaries (20). Alignment figures were rendered in ESPript 3.0 style using Python (v3.10.12) with Biopython (v1.87) and matplotlib (v3.10.8). Residue conservation was classified as strictly conserved (100% identity), physicochemically similar (based on Risler similarity groups), or non-conserved.

### DNA oligos used in this study

#### Amplify CAP5.5 from gDNA for protein expression

Forward: ATCACCATCACCATCACGGAATGGGCTGCTGCGCTTC
Reverse: CAACAGGAGTCCAAGCTCAGCTATTACTCCGCGTGGCCGT

#### Insert CAP5.5 guide RNA into Cas9 plasmid

Forward: GGCCACGCGGAGTAAATTGAgttttagagctagaaatagcaagtt
Reverse: GGATCCACTAGAACTCTTGCACAG

#### Tag CAP5.5 with Shark1

Forward: GATGACATTGCCGTTAAAATGAACGGCCACGCGGAGGAAGTGCATACTAATCAAGATCCC
Reverse: TCGTTCAGCAGCCCGTCGCCTCTCTCTCCCATACGCACGCttagccctcccacacataac

#### Amplify CAP5.5 from gDNA for Shark2-mediated ectopic expression

Forward: TGCTCTATAAGTTGTCTTGTCTAGAATGGGCTGCTGCGCTTC
Reverse: CGTTAAAATGAACGGCCACGCGGAGGGTaccGGGcccC

#### Screen CAP5.5 locus for recombination

Forward: CAAGGGAACATCGGAGGGAC
Reverse: CCCCGCTTCATTCACATCCT

#### Amplify CAP5.5-shark2 knock in cassette to replace CP2

Forward: CGAGGAATAGGGATAGGAACAAAGCTGAAGATGGGCTGCTGCGCTTC
Reverse: CACACACACGGAATAGTTACTGAAGAGTATggctcccggctttcgtg

#### Screen CP2 locus for recombination:

Forward: AGAAGCGCAAGGTTGAGGAA
Reverse: CAAACGCACACACACGGAAT

## SUPPLEMENTAL MATERIAL

Fig S1 msphere.00090-26-s0001.tif Production and characterization of anti-CAP5.5 antibodies.

Fig S2 msphere.00090-26-s0002.tif Multiple sequence alignment of CAP5.5 orthologs.

Fig S3 msphere.00090-26-s0003.tif Effect of Tet treatment on CAP5.5 abundance in Y-strain epimastigotes.

Fig S4 msphere.00090-26-s0004.tif Effect of Tet treatment on CAP5.5 abundance, cell proliferation, and kinetoplast/nucleus configuration in Y-strain amastigotes.

Fig S5 msphere.00090-26-s0005.tif Effect of rescue only CAP5.5 expression on epimastigote cell proliferation and kinetoplast/nucleus configuration.

ASM does not own the copyrights to Supplemental Material that may be linked to, or accessed through, an article. The authors have granted ASM a non-exclusive, world-wide license to publish the Supplemental Material files. Please contact the corresponding author directly for reuse.
