# Supplementary figures and images for: A dual-aptazyme system for genetic complementation reveals stage-specific roles for the Trypanosoma cruzi cytoskeleton-associated protein 5.5
Source: mSphere. 2026 May 29;11(6):e00090-26. doi: 10.1128/msphere.00090-26 (PMC13317235; doi:10.1128/msphere.00090-26)

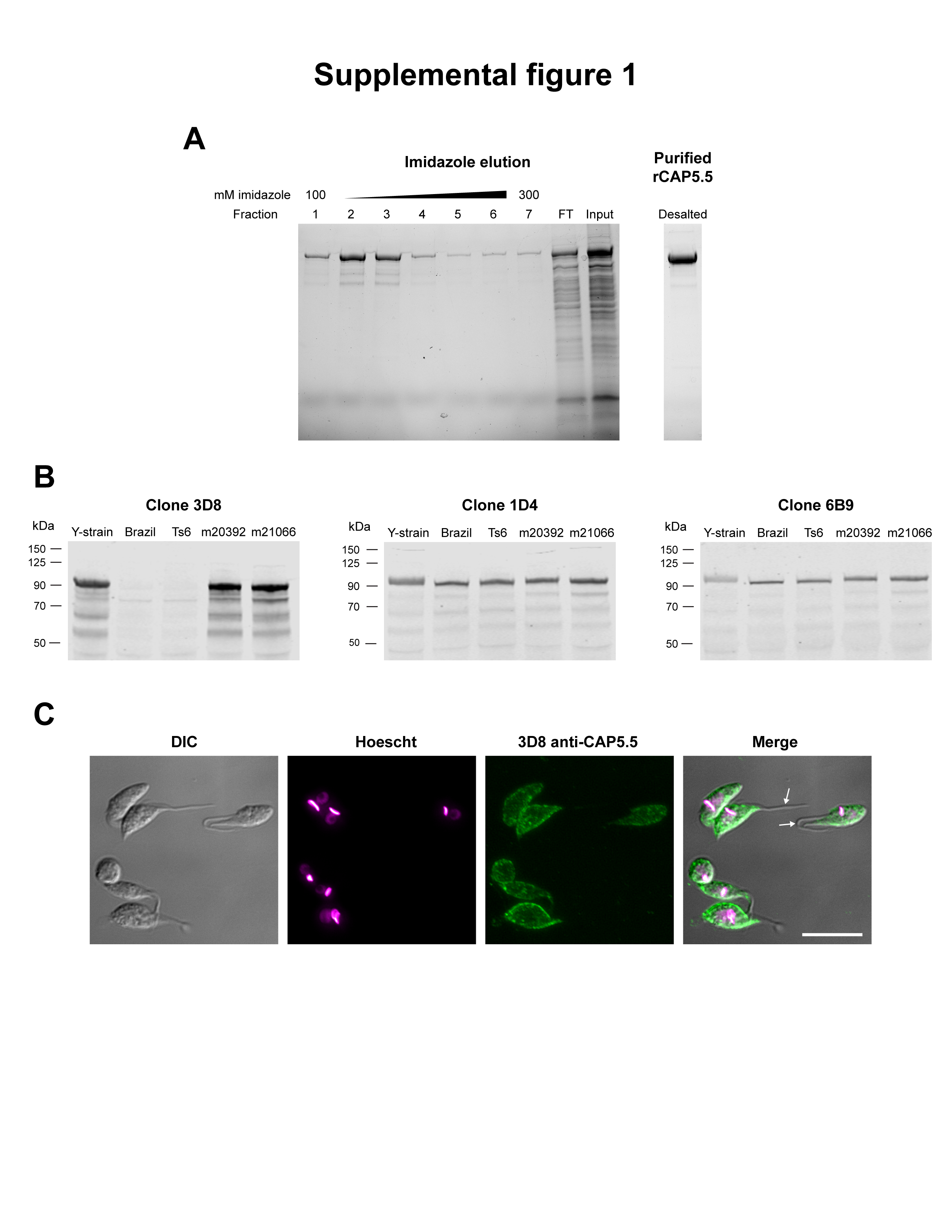

Supplement: Fig S1 — Production and characterization of anti-CAP5.5 antibodies. [file msphere.00090-26-s0001.tif]

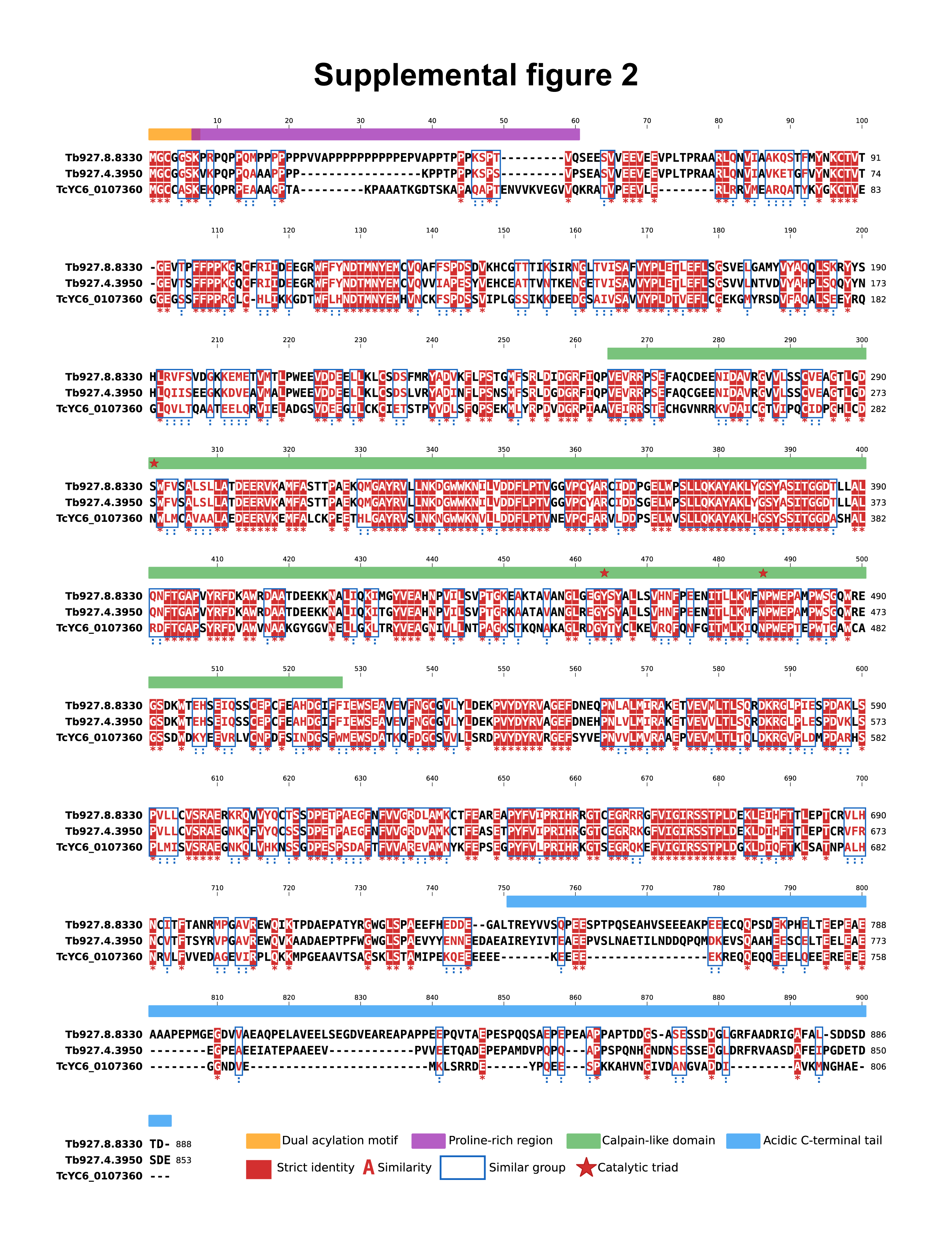

Supplement: Fig S2 — Multiple sequence alignment of CAP5.5 orthologs. [file msphere.00090-26-s0002.tif]

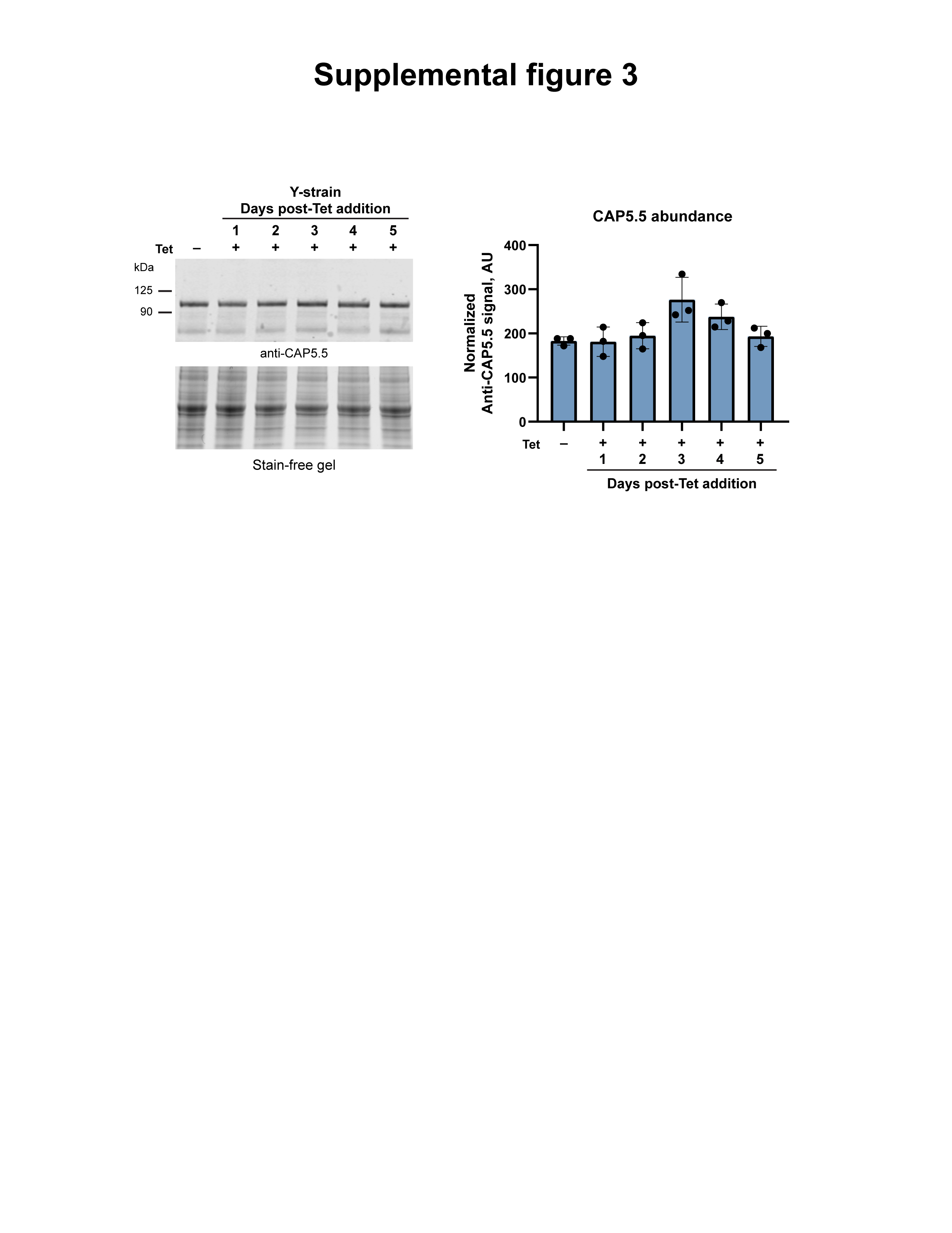

Supplement: Fig S3 — Effect of Tet treatment on CAP5.5 abundance in Y-strain epimastigotes. [file msphere.00090-26-s0003.tif]

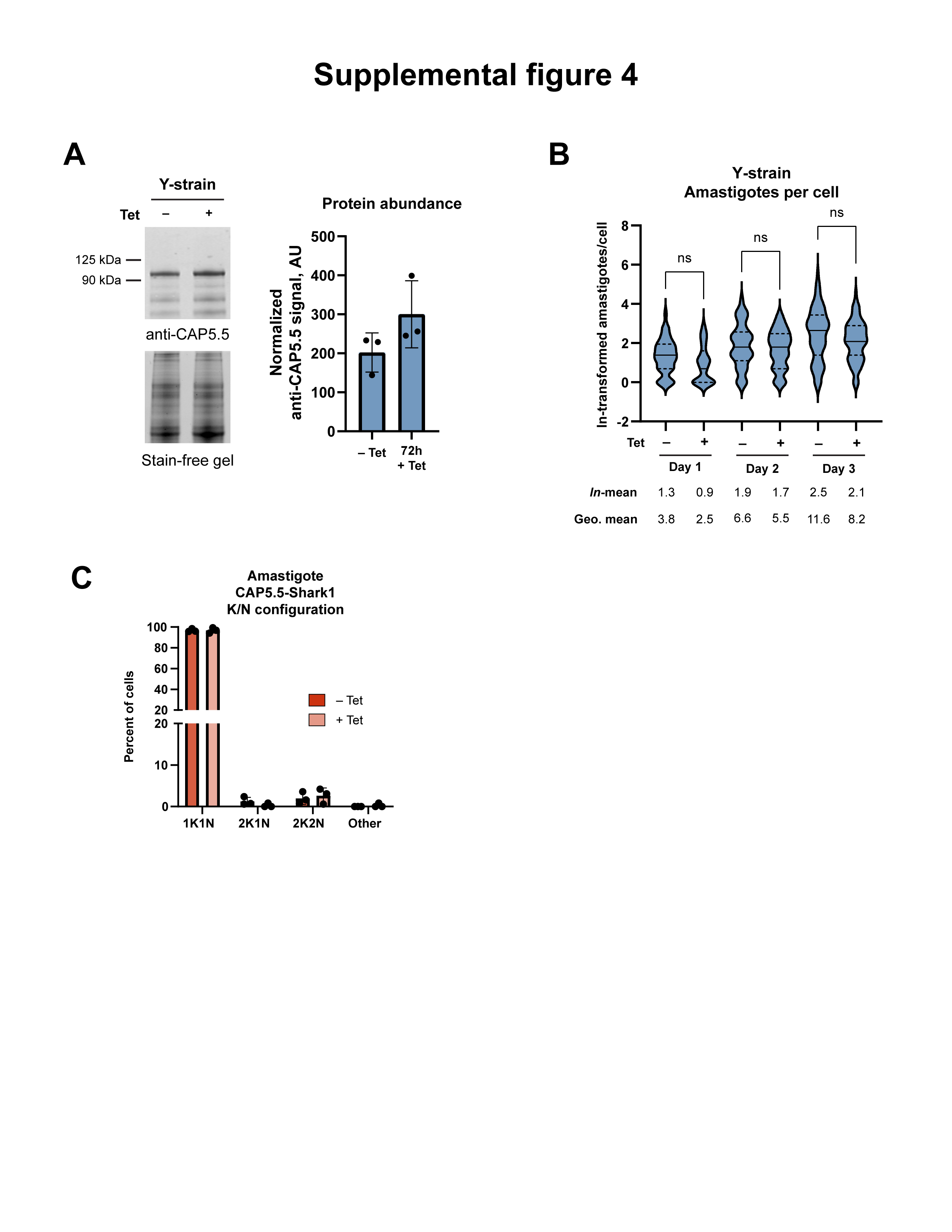

Supplement: Fig S4 — Effect of Tet treatment on CAP5.5 abundance, cell proliferation, and kinetoplast/nucleus configuration in Y-strain amastigotes. [file msphere.00090-26-s0004.tif]

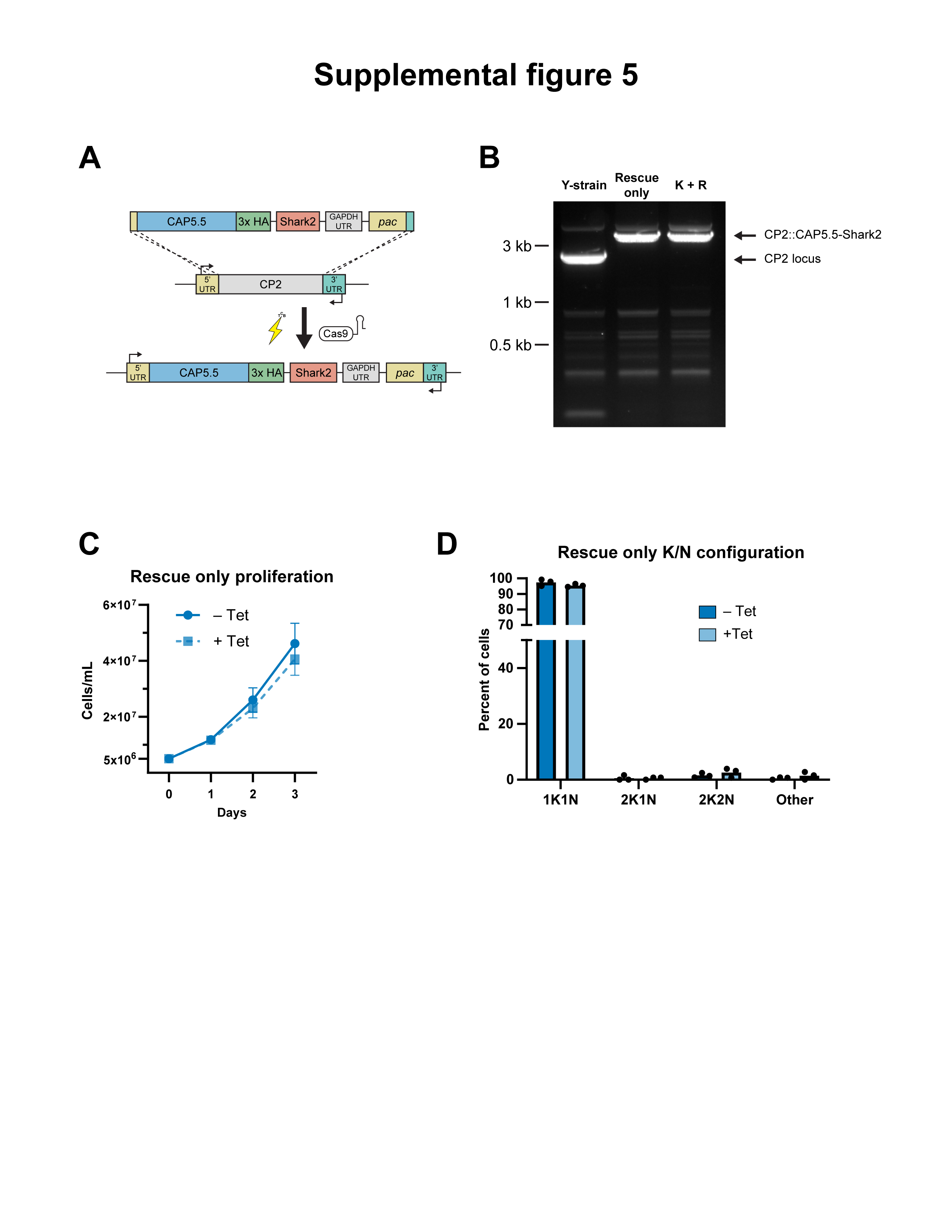

Supplement: Fig S5 — Effect of rescue only CAP5.5 expression on epimastigote cell proliferation and kinetoplast/nucleus configuration. [file msphere.00090-26-s0005.tif]
